# Reed & Carnrick

**Published:** 1882-02-20

**Authors:** 


REED & CARNRICK'S advertisement of Maltine should be carefully examined. The house is a reliable one, long established and all of their preparations of superior quality.

